# Supplementary material for: DENR controls JAK2 translation to induce PD-L1 expression for tumor immune evasion
Source: Nat Commun. 2022 Apr 19;13:2059. doi: 10.1038/s41467-022-29754-y (PMC9018773; doi:10.1038/s41467-022-29754-y)
Supplement: Supplementary file 1 — Supplementary information [file 41467_2022_29754_MOESM1_ESM.pdf]

Supplementary Information

DENR controls JAK2 translation to induce PD-L1 expression for tumor immune evasion

Baiwen Chen, Jiajia Hu, Xianting Hu, Huifang Chen, Rujuan Bao, Yatao Zhou, Youqiong Ye, Meixiao Zhan, Wei Cai, Huabin Li, Hua-Bing Li

1. Supplementary Tables..2
2. Supplementary Figures 5

1. Supplementary Tables

Supplementary Table 1: Antibodies used in this study

Antigen	Provider and Product Number	Blotting Dilution
DENR	10656-1-AP (Proteintech)	1:2000
PD-L1	ab213480 (Abcam)	1:1000
IFNGR1	10808-1-AP (Proteintech)	1:2000
IFNGR2	10266-1-AP (Proteintech)	1:2000
JAK1	50996S (Cell Signaling)	1:1000
P-JAK1	#SAB4300123 (Sigma)	1:1000
JAK2	3230S (Cell Signaling)	1:1000
P-JAK2	3771S (Cell Signaling)	1:1000
STAT1	14994S (Cell Signaling)	1:1000
P-STAT1	9167S (Cell Signaling)	1:1000
STAT3	12640 (Cell Signaling)	1:1000
P-STAT3	9131s (Cell Signaling)	1:1000
MCTS1	14984-1-AP (Proteintech)	1:1000
HA	ab9110 (Abcam)	1:2000
GAPDH	2118S (Cell Signaling)	1:5000
Actin	3700S (Cell Signaling)	1:5000
4E-BP1	9644T (Cell Signaling)	1:1000
P-4E-BP1	2855T (Cell Signaling)	1:1000
EEF2	ab75748 (Abcam)	1:1000
P-EEF2	ab82981 (Abcam)	1:1000
eIF2α	5324S (Cell Signaling)	1:1000
P-eIF2α	3398S (Cell Signaling)	1:1000
α/β-Tubulin	2148S (Cell Signaling)	1:5000
Anti-rabbit IgG HRP-linked antibody	7074P2 (Cell Signaling)	1:10000
HPR-conjugate Goat Anti-Mouse IgG	D110087-0025 (BBI Life Sciences)	1:10000
PE anti-mouse CD45.2	109808, Clone:104 (BioLegend)	1:250
PE/Cy7 anti-mouse CD8a	100722, Clone: 53-6.7 (BioLegend)	1:250
Alexa Fluor 700 anti-mouse CD4	100430, Clone: GK1.5 (BioLegend)	1:250

APC anti-mouse PD-L1	124312, Clone: 10F.9G2 (BioLegend)	1:250
PE anti-mouse PD-L1	124308, Clone: 10F.9G2 (BioLegend)	1:250
CellEvent™ Caspase-3/7 Green Detection Reagent	C10740 (ThermoFisher)	1:1000
LIVE/DEAD™ Fixable Near-IR Dead Cell Stain Kit	L10119 (ThermoFisher)	1:1000
Alexa Fluor 647 anti-P-STAT1	686412, Clone: A15158B (BioLegend)	1:250

Supplementary Table 2: sgRNA sequence

**Name**	**Sequence**
Denr sgRNA1	GAGAAGAAAAAACAAAAAAG
Denr sgRNA2	ATGTTGCTAAATGTAGACAA
Denr sgRNA3	TGAAGGCCAAGGACCAGTAG
Jak2 sgRNA	CATTAGCAAACTAAAGAAGG
Jak1 sgRNA	CAGGGTGAGCACCTTGGCAG
Stat1 sgRNA	GTGGCTGGGCGTCTATCCTG
Stat3 sgRNA	TCTCGTCCACCACCAAGCGG
Control sgRNA	GCGAGGTATTCGGCTCCGCG
PD-L1 sgRNA1	GCCTGCTGTCACTTGCTACG
PD-L1 sgRNA2	AATCAACCAGAGAATTTCCG
Brd2 sgRNA1	GAATGCAGGGACTTAAGAAG
Brd2 sgRNA2	CTTATAGAAGGGCCAGGCGT
Ndufa10 sgRNA1	GGGCTCACCTCATTTTGGGG
Ndufa10 sgRNA2	ATATAGATGACTGCGTGTGG
Atp5sl sgRNA1	CTGAGATGTCCAGCCTGCGG
Atp5sl sgRNA2	AGGTCTCACTCAGATCCTGG
Kat7 sgRNA1	ATGCCTCAGTACATGCGACA
Kat7 sgRNA2	AAAGCTTTGCCAAAAGGCAG
Human *DENR* sgRNA	ATGTTGCTAAATGTAGACAA

Supplementary Table 3: q-RT-PCR Primers

**Target**	**Sequence**
Jak2 forward	TGGCCTGCCTTACAATGACAG
Jak2 reverse	AAGGACTGGCTCTATCTGCTTC
Jak1 forward	GAGGACTGCAATGCCATGGC

Jak1 reverse	GGGGCTCCCTGTCCAACAGATA
Actb forward	GGCTGTATTCCCCTCCATCG
Actb reverse	CCAGTTGGTAACAATGCCATGT
PD-L1 forward	GCTCCAAAGGACTTGTACGTG
PD-L1 reverse	TGATCTGAAGGGCAGCATTTC
Ifngr1 forward	CTGGCAGGATGATTCTGCTGG
Ifngr1 reverse	GCATACGACAGGGTTCAAGTTAT
NF-κB forward	AGGCTTCTGGGCCTTATGTG
NF-κB reverse	TGCTTCTCTCGCCAGGAATAC
Stat1 forward	TCACAGTGGTTCGAGCTTCAG
Stat1 reverse	GCAAACGAGACATCATAGGCA
EGFP forward	ATGAAGCAGCACGACTTCTTCAA
EGFP reverse	GACGTTGTGGCTGTTGTAGTTGT
Gapdh forward	AGGTCGGTGTGAACGGATTTG
Gapdh reverse	TGTAGACCATGTAGTTGAGGTCA

2. Supplementary Figures

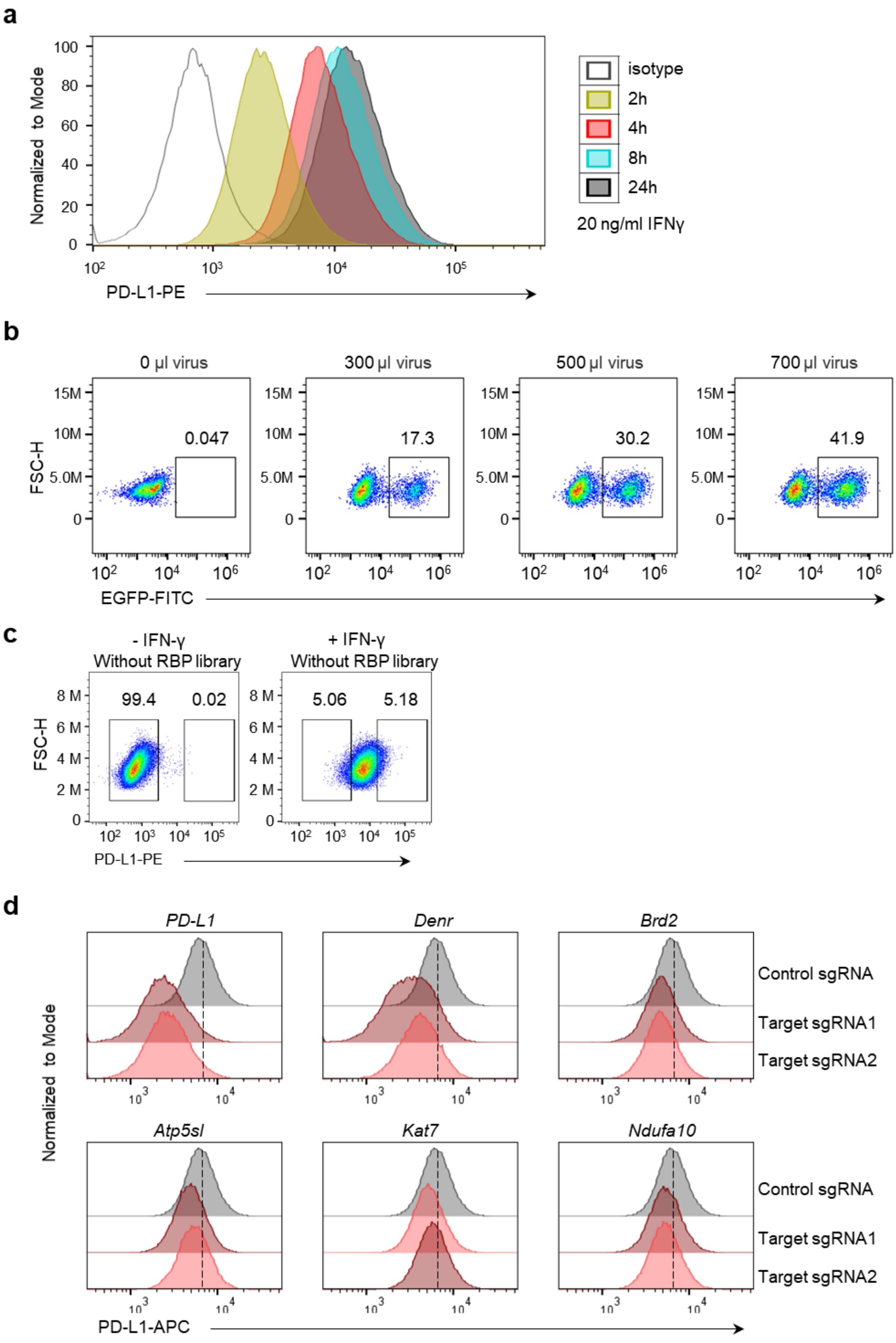

Supplementary Figure 1. PD-L1 expression of candidate KO cells.

a Flow cytometric analysis of PD-L1 expression on RAW264.7 cells treated with the indicated times of IFNγ. An IFNγ concentration of 20ng/ml for 4 h was chosen for the subsequent genetic screen, to allow identification of genes that either enhance or suppress PD-L1 expression. The experiment was repeated independently two times with similar results.

b The multiplicity of infection in RAW264.7 cells was tested with the EGFP-sgRNA plasmid. The experiment was repeated independently two times with similar results.

c Flow cytometric analysis of surface PD-L1 levels in RAW264.7 Cas9 expression cells without RBP sgRNAs library transfection. ± IFNγ (20 ng/ml) for 4 h. The experiment was repeated independently three times with similar results.

d Flow cytometric analysis of surface PD-L1 levels in RAW264.7 Cas9 expression cells transfected with identified top candidate sgRNAs, + IFNγ (20 ng/ml) for 8 h. The experiment was repeated independently two times with similar results. Source data are provided as a Source Data file.

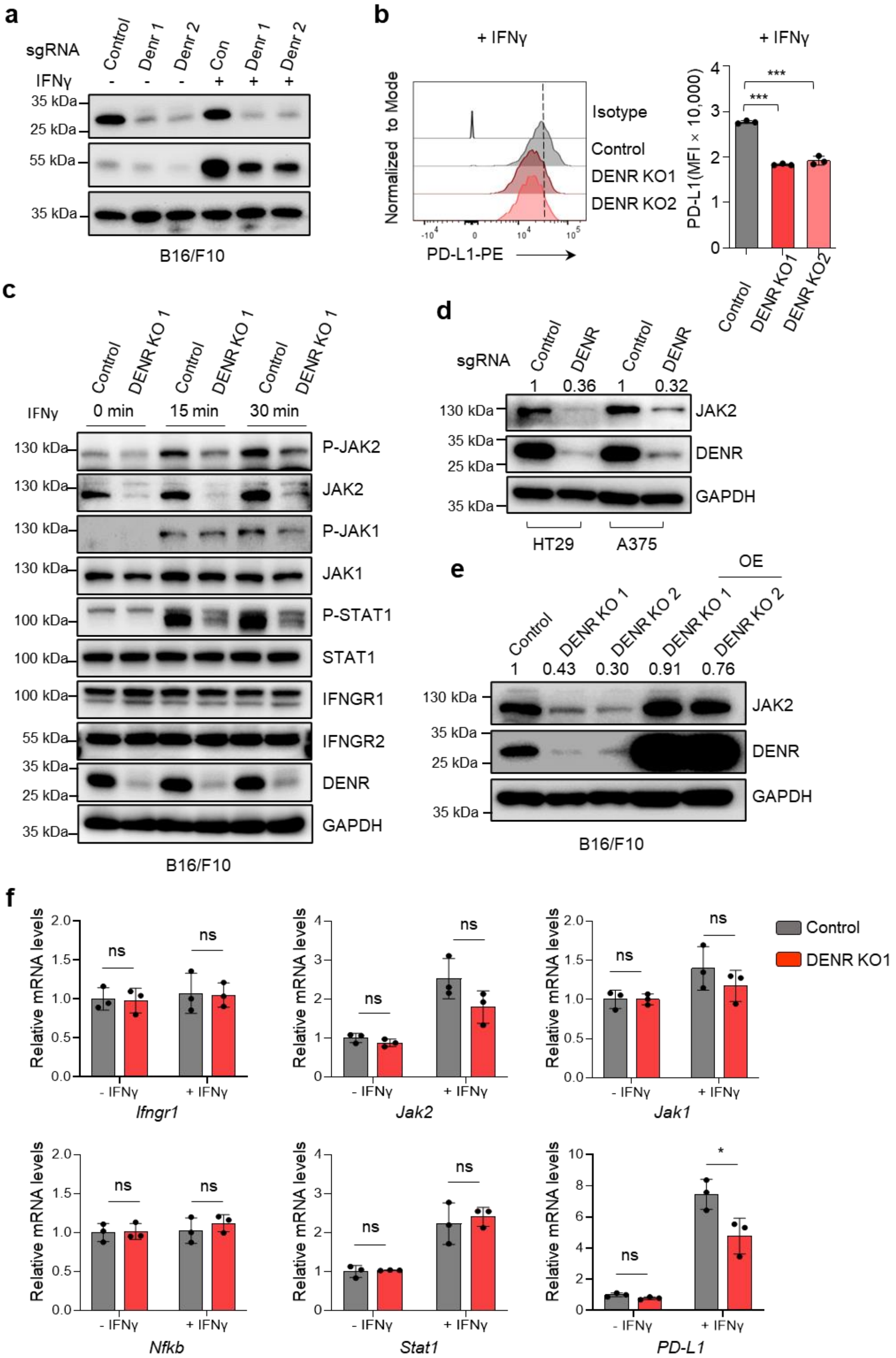

Supplementary Figure 2. DENR depletion of B16/F10 cells.

a Western blotting validation of DENR knockout and PD-L1 expression with two specific sgRNAs in B16/F10 cells, ± IFNγ (20 ng/ml) for 24 h. The experiment was repeated independently three times with similar results.

b Surface PD-L1 levels were analyzed by FACS in DENR and PD-L1 KO cells, + IFNγ (20 ng/ml) for 24 h (n = 3 independent samples). Results are representative of three biological replicates. Three technical replicates are shown. Data are presented as mean values ± SD. Two-sided, one-way ANOVA with Dunnett's post hoc test: *** $p < 0.001$. p values from left to right: < 0.0001, < 0.0001.

c Western blotting analysis of JAK1/p-JAK1, JAK2/p-JAK2, STAT1/p-STAT1, IFNGR1, and IFNGR2 in control or DENR KO B16/F10 cells. The experiment was repeated independently three times with similar results.

d Western blotting analysis of JAK2 in control or DENR KO cells of human colon carcinoma cell line HT29 and human melanoma cell line A375. The density of JAK2 bands were measured by normalizing to GAPDH by ImageJ. The experiment was repeated independently three times with similar results.

e sgRNA-resistance DENR cDNA expression vector was overexpressed in DENR KO B16/F10 cells, protein levels of JAK2 and DENR were measured 48 h later by western blotting. The density of JAK2 bands were quantified by normalizing to GAPDH by ImageJ. The experiment was repeated independently three times with similar results.

f RT-qPCR analysis of IFNγ signaling pathway in control and DENR KO B16/F10 cells, ± IFNγ (20 ng/ml) for 4 h (n = 3 biologically independent samples). Data are presented as mean values ± SD, unpaired two-tailed Student's t-test: ns, no significant, * $p < 0.05$. p values from left to right: 0.8735, 0.9031, 0.2253, 0.1294, 0.9982, 0.3188, 0.8922, 0.4489, 0.7337, 0.6297, 0.0704, 0.0364. Source data are provided as a Source Data file.

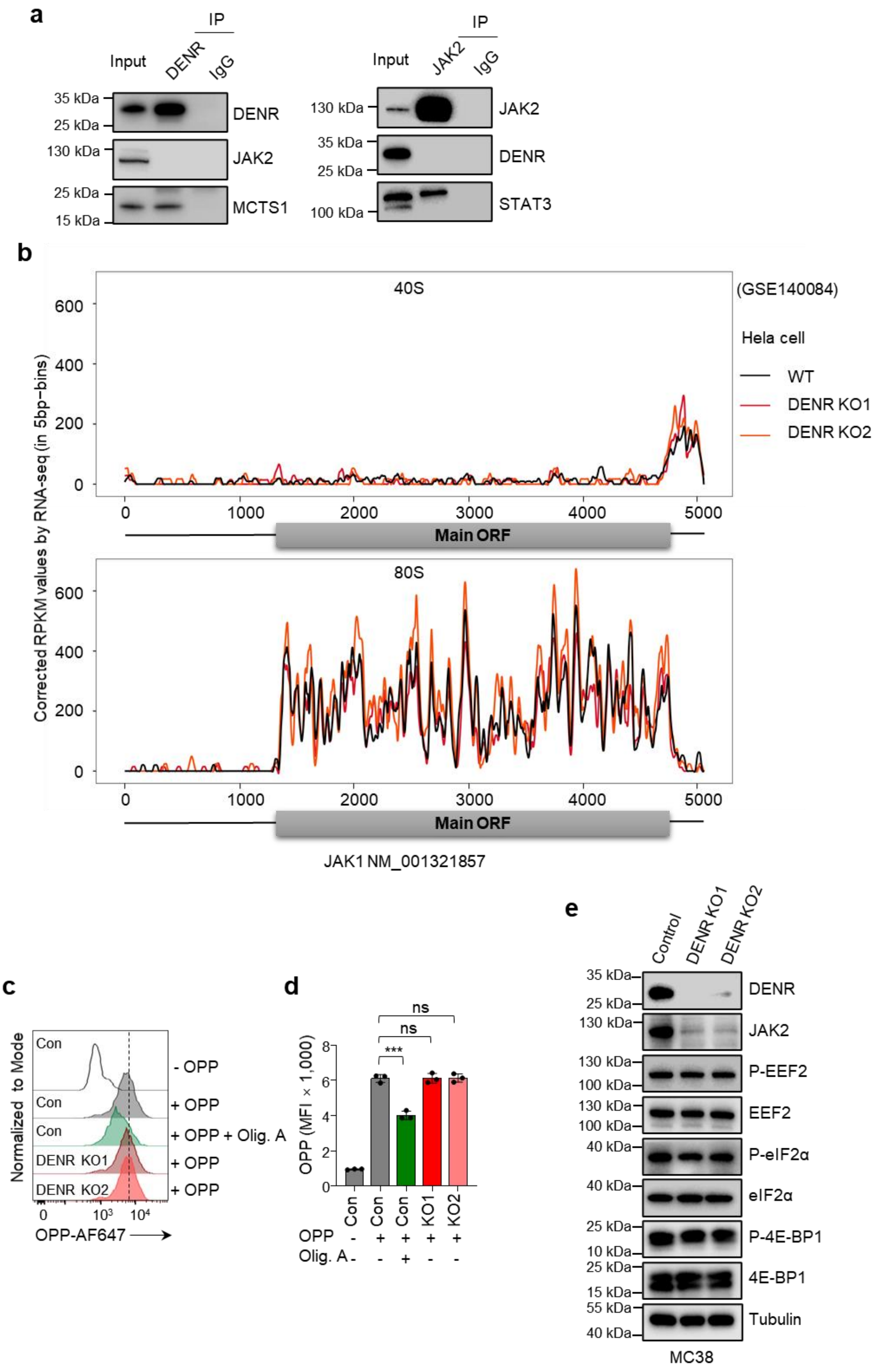

Supplementary Figure 3. DENR depletion does not effect on global translation.

a Anti-DENR or anti-JAK2 immunoprecipitation of MC38 cells. The experiment was

repeated independently three times with similar results.

b 40S and 80S ribosome occupancy of *JAK1* mRNA transcripts in DENR KO versus WT Hela cells.

c, d DENR was knocked out in MC38 cells by the LentiCRISPR v2-Blast. The OP-puro fluorescence was detected in control and DENR KO cells after labeling with OP-puro for 30 min (n = 3 independent samples). Oligomycin A (3 ng/ml) was added for 3 hours before OP-puro to suppress the activity of translation as a positive control. Results are representative of three biological replicates. Three technical replicates are shown. Data are presented as mean values ± SD. Two-sided, one-way ANOVA with Dunnett's post hoc test: ns, no significant, *** $p < 0.001$. p values from left to right: < 0.0001, <0.0001, > 0.9999, 0.9993.

e Western blotting analysis of the phosphorylation status of eIF2a, 4EBP1, eEF2 in the control and DENR KO MC38 cells. The experiment was repeated independently three times with similar results. Source data are provided as a Source Data file.

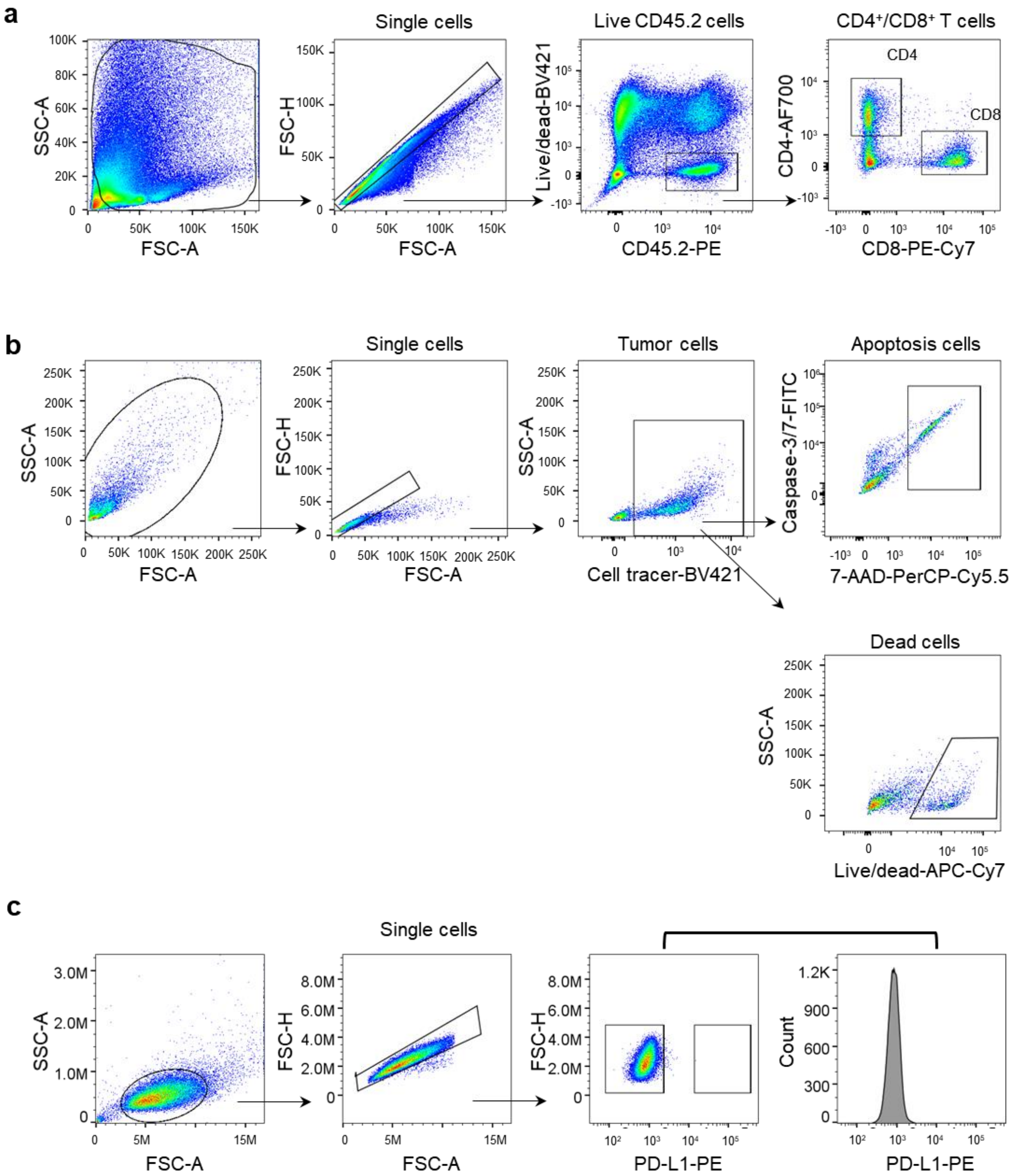

Supplementary Figure 4. Flow cytometry gating strategies used in the study.

a Gating strategy as shown in Figure 6f. Isolated cells in tumor tissues were gated for single live $CD45^+$ cells. $CD4^+$ and $CD8^+$ T cells were identified form live $CD45^+$ T cells

b Gating strategy for apoptosis or dead tumor cells from co-cultured $CD8^+$ T cells as shown in Figure 7a Supplementary Figure 5e.

c Gating strategy for PD-L1 expression in this study and CRISPR screening as shown in Figure 1b, 1e, 2b, 3e, Supplementary Figure 2b. Source data are provided as a Source Data file.

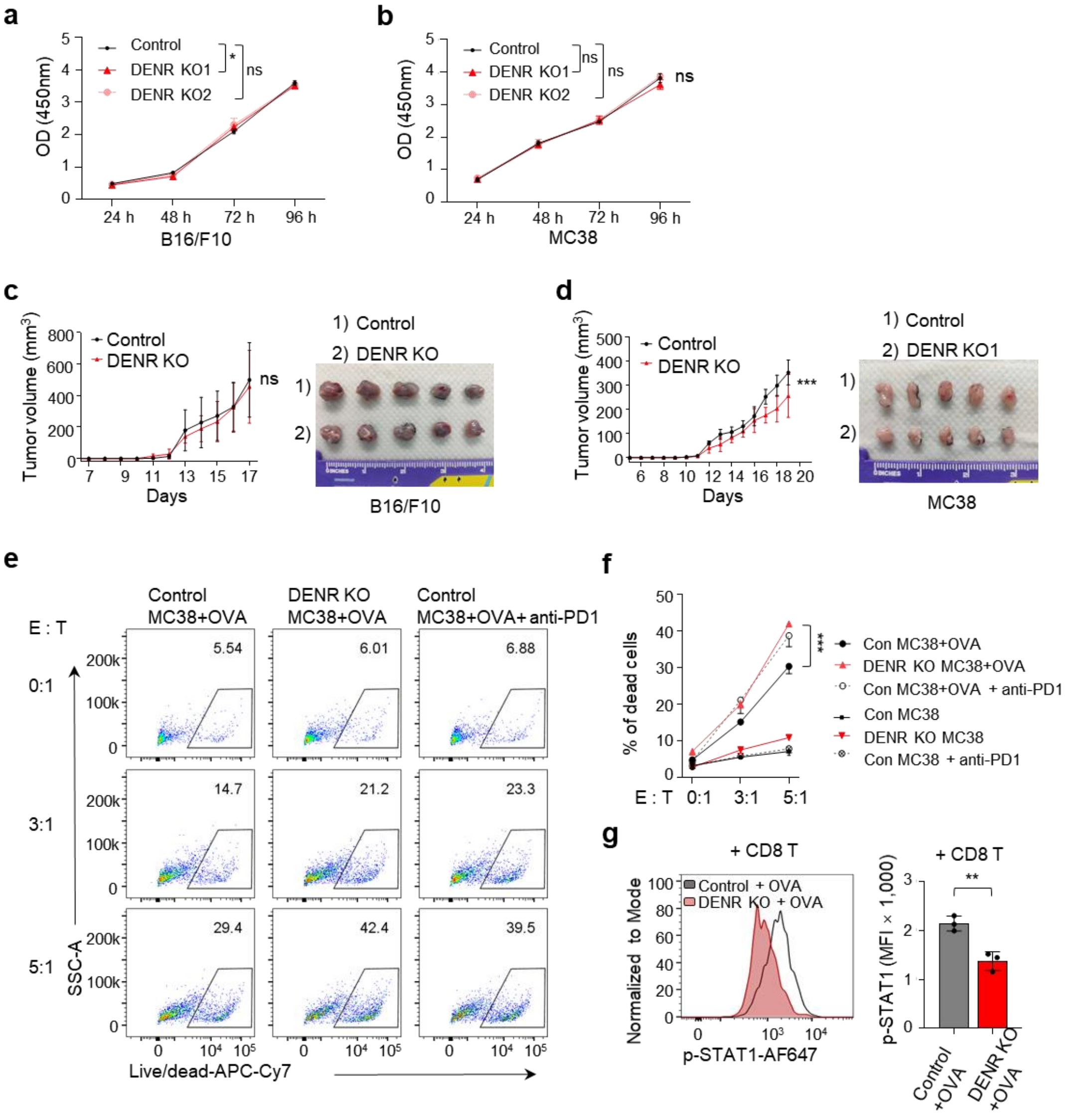

Supplementary Figure 5. DENR depletion does not strongly suppress the tumor growth in immunodeficiency mice.

a, b CCK8 assay was performed in control or DENR KO cells, 500 B16/F10 cells or 1,000 MC38 cells were culture in 96-wells plate, the OD (450nm) values were measured at the indicated time point (n = 3 independent samples). Results are representative of three biological replicates. Three technical replicates are shown. Data are presented as mean values ± SD, two-way ANOVA test: ns, no significant, * $p < 0.05$. p values from left to right: 0.0185, 0.0563, 0.0946, 0.7555.

c, d Female RAG1$^{-/-}$ mice (6-8 weeks) were given 1×10^5 control or DENR KO cells, and tumors were measured every day (n = 5 mice). Tumor growth is representative of two independent experiments, with at least four mice per group. Data are presented as mean values ± SD, two-way ANOVA test: ns, no significant, *** $p < 0.001$. p values from left to right: > 0.9999, < 0.0001.

e, f Control or DENR KO MC38 cells with or without expression OVA were co-cultured with OT-1 $CD8^+$ T cells at an effector to target ratio of 0:1, 3:1 or 5:1 for 16 h; anti-PD-1 (10 µg/ml) was added as a positive control. The global cell death was gated as in **e** and quantified as in **f**. The apoptosis cells were measured by flow cytometry (n = 3 independent samples). Results are representative of three biological replicates. Three technical replicates are shown. Data are presented as mean values ± SD, unpaired two-tailed Student's t-test at E : T = 5:1 : *** $p < 0.001$. $p = 0.0007$.

g Control or DENR KO MC38 cells with expression OVA were incubated with OT-1 $CD8^+$ T cells at an effector to target ratio of 3:1 for 3 h and intracellular p-STAT1 was measured by flow cytometry (n = 3 independent samples). Results are representative of three biological replicates. Three technical replicates are shown. Data are presented as mean values ± SD, unpaired two-tailed Student's t-test: ** $p < 0.01$. $p = 0.0056$. Source data are provided as a Source Data file.

Supplementary Figure 2a

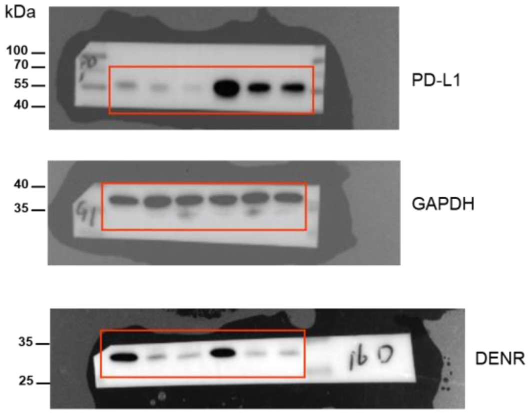

Supplementary Figure 2c

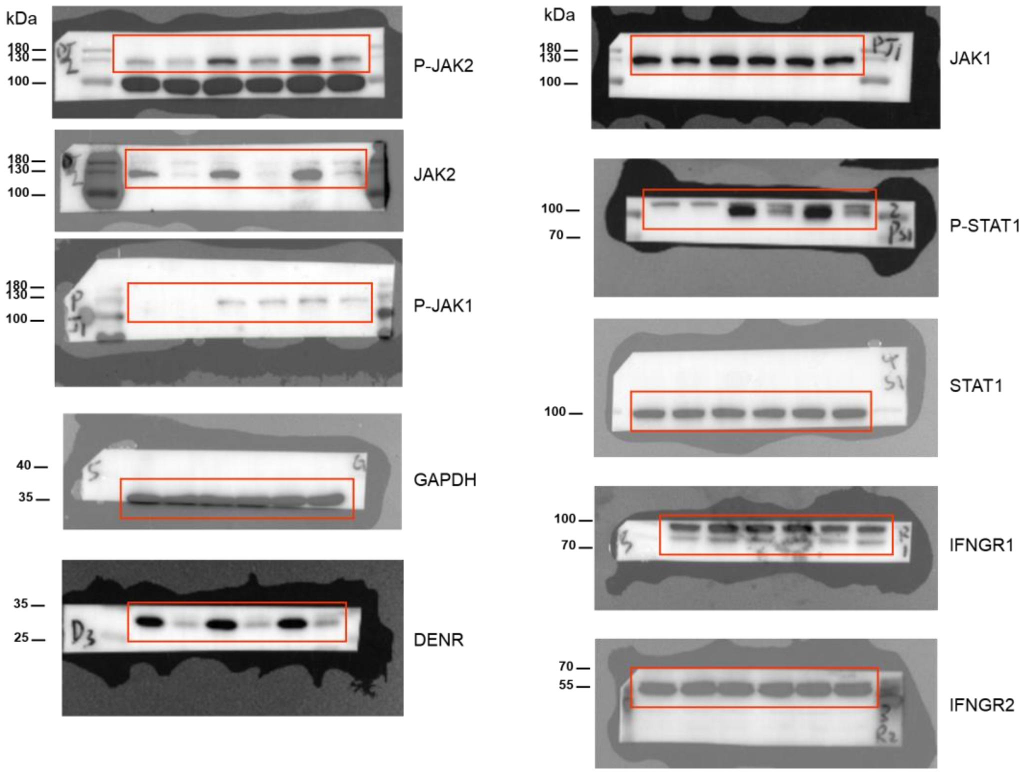

Supplementary Figure 2d

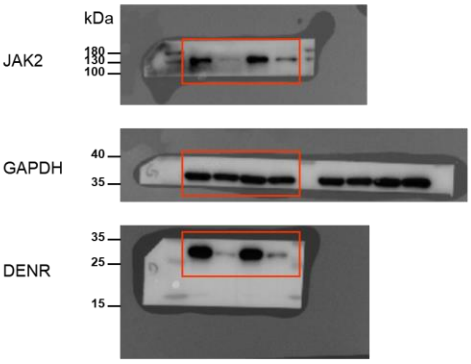

Supplementary Figure 2e

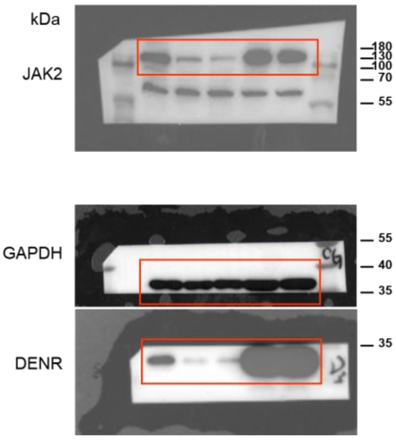

Supplementary Figure 3a

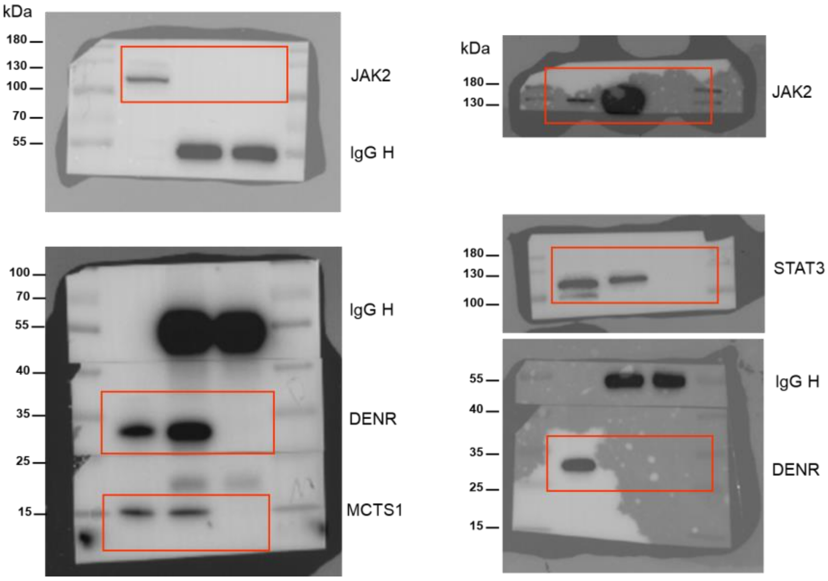

Supplementary Figure 3e

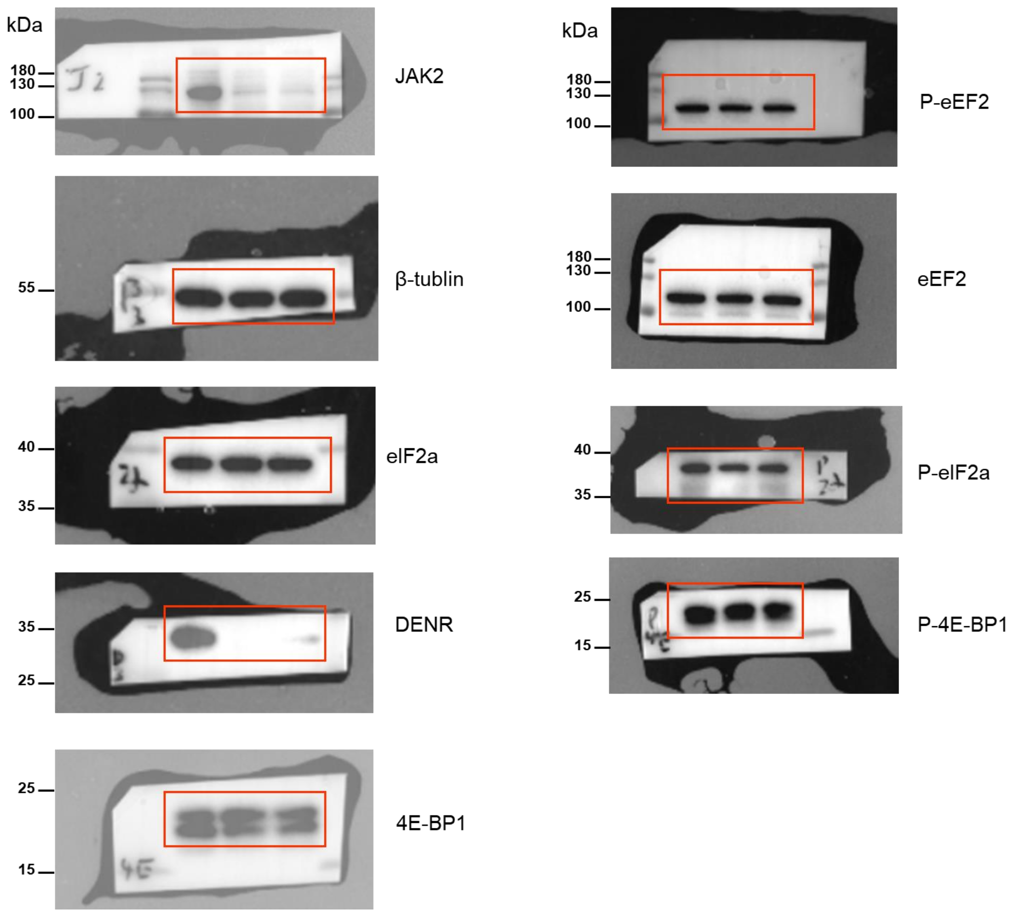

Supplementary Figure 6. Uncropped blots. Related to Supplementary Figures 2, 3.
