# Supplementary material for: DENR controls JAK2 translation to induce PD-L1 expression for tumor immune evasion
Source: Nat Commun. 2022 Apr 19;13:2059. doi: 10.1038/s41467-022-29754-y (PMC9018773; doi:10.1038/s41467-022-29754-y)
Supplement: Supplementary file 2 — Reporting Summary [file 41467_2022_29754_MOESM2_ESM.pdf]

nature portfolio

Corresponding author(s): Hua-Bing Li

Last updated by author(s): Mar 22, 2022

Reporting Summary

Nature Portfolio wishes to improve the reproducibility of the work that we publish. This form provides structure for consistency and transparency in reporting. For further information on Nature Portfolio policies, see our Editorial Policies and the Editorial Policy Checklist.

Statistics

For all statistical analyses, confirm that the following items are present in the figure legend, table legend, main text, or Methods section.

n/a	Confirmed	
☐	☒	The exact sample size (n) for each experimental group/condition, given as a discrete number and unit of measurement
☐	☒	A statement on whether measurements were taken from distinct samples or whether the same sample was measured repeatedly
☐	☒	The statistical test(s) used AND whether they are one- or two-sided<br>*Only common tests should be described solely by name; describe more complex techniques in the Methods section.*
☒	☐	A description of all covariates tested
☐	☒	A description of any assumptions or corrections, such as tests of normality and adjustment for multiple comparisons
☐	☒	A full description of the statistical parameters including central tendency (e.g. means) or other basic estimates (e.g. regression coefficient) AND variation (e.g. standard deviation) or associated estimates of uncertainty (e.g. confidence intervals)
☐	☒	For null hypothesis testing, the test statistic (e.g. F, t, r) with confidence intervals, effect sizes, degrees of freedom and P value noted<br>*Give P values as exact values whenever suitable.*
☒	☐	For Bayesian analysis, information on the choice of priors and Markov chain Monte Carlo settings
☒	☐	For hierarchical and complex designs, identification of the appropriate level for tests and full reporting of outcomes
☒	☐	Estimates of effect sizes (e.g. Cohen's d, Pearson's r), indicating how they were calculated

Our web collection on statistics for biologists contains articles on many of the points above.

Software and code

Policy information about availability of computer code

Data collection	Raw data were analyzed by FlowJo V10 for flow cytometric data, by ImageJ 1.52a for immunofluorescence, by HiSeq 2000 System for RNA-seq data, by Survminer_0.4.9 package for survival information.
Data analysis	Statistical analyses were performed with Graphpad Prism 8.

For manuscripts utilizing custom algorithms or software that are central to the research but not yet described in published literature, software must be made available to editors and reviewers. We strongly encourage code deposition in a community repository (e.g. GitHub). See the Nature Portfolio guidelines for submitting code & software for further information.

Data

Policy information about availability of data

All manuscripts must include a data availability statement. This statement should provide the following information, where applicable:

- Accession codes, unique identifiers, or web links for publicly available datasets
- A description of any restrictions on data availability
- For clinical datasets or third party data, please ensure that the statement adheres to our policy

The accession number for the RNA-seq is GSE183980 [https://www.ncbi.nlm.nih.gov/geo/query/acc.cgi?acc=GSE183980], and for CRISPR screen is GSE184048 [https://www.ncbi.nlm.nih.gov/geo/query/acc.cgi?acc=GSE184048]. For GSEA analysis the gene set “c2.all.v7.5” was downloaded from the MSigDB database [http://www.gsea-msigdb.org/gsea/index.jsp]. All other remaining data are available within the Article and Supplementary Files.

Field-specific reporting

Please select the one below that is the best fit for your research. If you are not sure, read the appropriate sections before making your selection.

☒ Life sciences ☐ Behavioural & social sciences ☐ Ecological, evolutionary & environmental sciences

For a reference copy of the document with all sections, see nature.com/documents/nr-reporting-summary-flat.pdf

Life sciences study design

All studies must disclose on these points even when the disclosure is negative.

Sample size	Sample size is indicated in the figure legend for each experiments. No sample size calculation was done either for in vivo or in vitro studies. For in vivo studies, n=5 mice per group is sufficient to detect meaningful biological differences with good reproducibility. For cell-based in vitro experiments, results of two or three independent biological replicates were used.
Data exclusions	No data were excluded in this study.
Replication	Data shown are representative results of at least two independent experiments with similar results. Consistent difference (or no difference) was observed, as indicated by the average value of each group. All attempts at replication were successful.
Randomization	Mice were randomly allocated into each group. For cell-based experiments, samples were randomly allocated to each group, and subsequently handled and processed identically.
Blinding	Data were collected by the same person carrying out the expriments. Three major authors worked individually and collaboratively on the project. Therefore, the data were not collected in a blinded manner.

Reporting for specific materials, systems and methods

We require information from authors about some types of materials, experimental systems and methods used in many studies. Here, indicate whether each material, system or method listed is relevant to your study. If you are not sure if a list item applies to your research, read the appropriate section before selecting a response.

Materials & experimental systems

n/a	Involved in the study	
☐	☒	Antibodies
☐	☒	Eukaryotic cell lines
☒	☐	Palaeontology and archaeology
☐	☒	Animals and other organisms
☒	☐	Human research participants
☒	☐	Clinical data
☒	☐	Dual use research of concern

Methods

n/a	Involved in the study	
☒	☐	ChIP-seq
☐	☒	Flow cytometry
☒	☐	MRI-based neuroimaging

Antibodies

Antibodies used	DENR 10656-1-AP (Proteintech), PD-L1 ab213480 (Abcam), IFNGR1 10808-1-AP (Proteintech), IFNGR2 10266-1-AP (Proteintech), JAK1 50996S (Cell Signaling), P-JAK1 #ASB4300123 (Sigma), JAK2 3230S (Cell Signaling), P-JAK2 3771S (Cell Signaling), STAT1 14994S (Cell Signaling), P-STAT1 9167S (Cell Signaling), STAT3 12640 (Cell Signaling), P-STAT3 9131s (Cell Signaling), MCTS1 14984-1-AP (Proteintech), HA ab9110 (Abcam), GAPDH 2118S (Cell Signaling), Actin 3700S (Cell Signaling), 4E-BP1 9644T (Cell Signaling), P-4E-BP1 2855T (Cell Signaling), EEF2 ab75748 (Abcam), P-EEF2 ab82981 (Abcam), eIF2α 5324S (Cell Signaling), P-eIF2α 3398S (Cell Signaling), α/β-Tubulin 2148S (Cell Signaling), Anti-rabbit IgG HRP-linked antibody 7074P2 (Cell Signaling), HPR-conjugate Goat Anti-Mouse IgG, D110087-0025 (BBI Life Sciences), PE anti-mouse CD45.2 Clone:104 (BioLegend), PE/Cy7 anti-mouse CD8a Clone: 53-6.7 (BioLegend), Alexa Fluor 700 anti-mouse CD4 Clone: GK1.5 (BioLegend), APC anti-mouse PD-L1 Clone: 10F.9G2 (BioLegend), PE anti-mouse PD-L1, Clone: 10F.9G2 (BioLegend), CellEvent™ Caspase-3/7 Green Detection Reagent C10740 (ThermoFisher), LIVE/DEAD™ Fixable Near-IR, Dead Cell Stain Kit L10119 (ThermoFisher), Alexa Fluor 647 anti-P-STAT1 Clone: A15158B (BioLegend).
Validation	All the primary antibodies used in this study have been validated in mouse for WB or FACS by the manufactures. The primary antibodies of PD-L1, JAK1, JAK2, STAT1, STAT3, DENR has been validated by knock out cell lines in the study.<br>DENR Polyclonal antibody, Reactivity: Human, Mouse, Rat. Applications: WB, IF. [https://www.ptglab.com/products/DENR-Antibody-10656-1-AP.htm].PMID: 33752661<br>Anti-PD-L1, Reactivity: Mouse. Applications: ICC/IF, IP, WB. [https://www.abcam.cn/pd-l1-antibody-epr20529-ab213480.html].PMID: 33390170<br>IFNGR1 Polyclonal antibody, Reactivity: Human, Mouse. Applications: WB, IP, IHC, FC, ELISA. [https://www.ptglab.com/products/IFNGR1-Antibody-10808-1-AP.htm].PMID: 28174303

IFNGR2 Polyclonal antibody, Reactivity: Human, Mouse. Applications: WB, IP, IHC, FC, ELISA. [https://www.ptglab.com/products/IFNGR2-Antibody-10266-1-AP.htm]. Pubmed ID: 28562647
JAK1, Reactivity: Human, Mouse, Rat. Applications: WB, IP. [https://www.cellsignal.com/products/primary-antibodies/jak1-d1t6w-mouse-mab/50996?site-search-type=Products&N=4294956287&Ntt=50996s&fromPage=plp&_requestid=4426936&country=USA]. PMID: 35024200
p-JAK1, Reactivity: Human, Mouse, Rat. Applications: WB, IHC. [https://www.sigmaaldrich.cn/CN/en/specification-sheet/SIGMA/SAB4300123]. PMID: 29055957
JAK2, Reactivity: Human, Mouse, Rat. Applications: WB, IP, IF. [https://www.cellsignal.com/products/primary-antibodies/jak2-d2e12-xp-rabbit-mab/3230?site-search-type=Products&N=4294956287&Ntt=3230s&fromPage=plp&_requestid=4281351]. PMID: 34738622
p-JAK2, Reactivity: Human, Mouse. Applications: WB, IP. [https://www.cellsignal.com/products/primary-antibodies/phospho-jak2-tyr1007-1008-antibody/3771?_=1647928802873&Ntt=3771S&tahead=true]. PMID: 34725437
STAT1, Reactivity: Human, Mouse. Applications: WB, IP, IHC, IF, F, ChIP. [https://www.cellsignal.com/products/primary-antibodies/stat1-d1k9y-rabbit-mab/14994?site-search-type=Products&N=4294956287&Ntt=14994s&fromPage=plp&_requestid=4282524]. PMID: 34420035
p-STAT1, Reactivity: Human, Mouse. Applications: WB, IP, IHC, IF, F, ChIP. [https://www.cellsignal.com/products/primary-antibodies/phospho-stat1-tyr701-58d6-rabbit-mab/9167?site-search-type=Products&N=4294956287&Ntt=9167s&fromPage=plp&_requestid=4282862]. PMID: 34992585
STAT3, Reactivity: Human, Mouse, Rat, Monkey. Applications: WB, IP, IF, F, ChIP. [https://www.cellsignal.com/products/primary-antibodies/stat3-d3z2g-rabbit-mab/12640]. PMID: 34976194
p-STAT3, Reactivity: Human, Mouse, Rat, Monkey. Applications: WB, IP, ChIP. [https://www.cellsignal.com/products/primary-antibodies/phospho-stat3-tyr705-antibody/9131?site-search-type=Products&N=4294956287&Ntt=9131s&fromPage=plp&_requestid=4284734]. PMID: 34990523
MCTS1, Reactivity: Human, Mouse, Rat. Applications: WB, ELISA. [https://www.ptglab.com/products/MCTS1-Antibody-14984-1-AP.htm].
Anti-HA tag, Reactivity: Species independent. Applications: ChIP, IP, ELISA, WB, ICC/IF, Flow Cyt. [https://www.abcam.cn/ha-tag-antibody-chip-grade-ab9110.html]. PMID: 32077934
GAPDH, Reactivity: Human, Mouse, Rat, Monkey, Bovine, Pig. Applications: WB, IHC, IF, F. [https://www.cellsignal.com/products/primary-antibodies/gapdh-14c10-rabbit-mab/2118?site-search-type=Products&N=4294956287&Ntt=2118s&fromPage=plp&_requestid=4303749]. PMID: 35024243
Actin, Reactivity: Human, Mouse, Rat, Hamster, Monkey, Dog. Applications: WB, IHC, IF, F. [https://www.cellsignal.com/products/primary-antibodies/b-actin-8h10d10-mouse-mab/3700?site-search-type=Products&N=4294956287&Ntt=3700s+&fromPage=plp&_requestid=4304427]. PMID: 34977268
4E-BP1, Reactivity: Human, Mouse, Rat, Monkey. Applications: WB, IP, IHC, IF, F. [https://www.cellsignal.com/products/primary-antibodies/4e-bp1-53h11-rabbit-mab/9644?site-search-type=Products&N=4294956287&Ntt=9644t&fromPage=plp&_requestid=4305210]. PMID: 35005567
p-4E-BP1, Reactivity: Human, Mouse, Rat, Monkey, D. melanogaster. Applications: WB, IHC, IF, F. [https://www.cellsignal.com/products/primary-antibodies/phospho-4e-bp1-thr37-46-236b4-rabbit-mab/2855?site-search-type=Products&N=4294956287&Ntt=2855t&fromPage=plp&_requestid=4305806]. PMID: 34977433
EEF2, Reactivity: Mouse, Rat, Human. Applications: Flow Cyt (Intra), WB, IP, IHC-P, ICC/IF. [https://www.abcam.cn/eef2elongation-factor-2-antibody-ep880y-ab75748.html]. PMID: 32899302
p-EEF2, Reactivity: Human. Predictions can be used to Mouse, Rat. Applications: WB. [https://www.abcam.cn/eef2elongation-factor-2-phospho-t56--t58-antibody-ep833y-ab82981.html]. PMID: 31619542
eIF2α, Reactivity: Human, Mouse, Rat, Monkey. Applications: WB, IP, IHC. [https://www.cellsignal.com/products/primary-antibodies/eif2a-d7d3-xp-rabbit-mab/5324?site-search-type=Products&N=4294956287&Ntt=5324s&fromPage=plp&_requestid=4308917]. PMID: 35013318
p-eIF2α, Reactivity: Human, Mouse, Rat, Monkey, D. melanogaster. Applications: WB, IP, IHC. [https://www.cellsignal.com/products/primary-antibodies/phospho-eif2a-ser51-d9g8-xp-rabbit-mab/3398?site-search-type=Products&N=4294956287&Ntt=3398s&fromPage=plp&_requestid=4309468]. PMID: 34977370
α/β-Tubulin, Reactivity: Human, Mouse, Rat, Monkey, Zebrafish, Bovine. Applications: WB, IF, F, IHC. [https://www.cellsignal.com/products/primary-antibodies/a-b-tubulin-antibody/2148?site-search-type=Products&N=4294956287&Ntt=2148s&fromPage=plp&_requestid=4309838]. PMID: 35003516
PE anti-mouse CD45.2, Reactivity: Mouse. Application: FC. [https://www.biolegend.com/en-us/products/pe-anti-mouse-cd45-2-antibody-7]. PMID: 32579887
PE/Cy7 anti-mouse CD8a, Reactivity: Mouse. Application: FC. [https://www.biolegend.com/en-us/products/pe-cyanine7-anti-mouse-cd8a-antibody-1906]. PMID: 26728942
Alexa Fluor 700 anti-mouse CD4, Reactivity: Mouse. Application: FC. [https://www.biolegend.com/en-us/products/alexa-fluor-700-anti-mouse-cd4-antibody-3385]. PMID: 30455700
APC anti-mouse PD-L1, Reactivity: Mouse. Application: FC. [https://www.biolegend.com/en-us/products/apc-anti-mouse-cd274-b7-h1-pd-l1-antibody-6655]. PMID: 29548673
PE anti-mouse PD-L1, Reactivity: Mouse. Application: FC. [https://www.biolegend.com/en-us/products/pe-anti-mouse-cd274-b7-h1-pd-l1-antibody-4497]. PMID: 31851488
Alexa Fluor 647 anti-P-STAT1, Reactivity: Mouse. Application: ICFC. [https://www.biolegend.com/en-us/products/alexa-fluor-647-anti-stat1-phospho-ser727-antibody-14229]. PMID: 32853638

Eukaryotic cell lines

Policy information about cell lines

Cell line source(s) | HEK293T, RAW264.7, MC38, B16/F10, HT29 and A375 cells were from American Type Culture Collection (ATCC).

Authentication	No authentication was carried out. Cell morphology and behavior such as tumor growth was consistent with expectations.
Mycoplasma contamination	Cell lines were tested negative for mycoplasma contamination.
Commonly misidentified lines (See ICLAC register)	No misidentified lines were used.

Animals and other organisms

Policy information about studies involving animals; ARRIVE guidelines recommended for reporting animal research

Laboratory animals	Female WT or RAG1-/- C57BL/6 mice (6-8 weeks) were purchased from Shanghai Model Organisms Center.
Wild animals	The study did not involve wild animals.
Field-collected samples	The study did not involve samples collected from the field.
Ethics oversight	The animals were maintained in specific-pathogen-free facilities and used according to protocols approved by Animal Care and Use Committees of the SJTUSM.

Note that full information on the approval of the study protocol must also be provided in the manuscript.

Flow Cytometry

Plots

Confirm that:

- [x] The axis labels state the marker and fluorochrome used (e.g. CD4-FITC).
- [x] The axis scales are clearly visible. Include numbers along axes only for bottom left plot of group (a 'group' is an analysis of identical markers).
- [x] All plots are contour plots with outliers or pseudocolor plots.
- [x] A numerical value for number of cells or percentage (with statistics) is provided.

Methodology

Sample preparation	Tumors were cut into small pieces before digested in RPMI-1640 medium with 1 mg/ml type IV collagenase and 100 ug/ml DNase I. After digestion, tissues were passed through a 70 um cell strainer to make single cell, suspensions. Then cells were incubated with antibodies for 30 min. After washed, samples were analyzed on a flow cytometer.
Instrument	BD LSRFortessa X-20 or Accuri C6 Plus.
Software	FlowJo v10
Cell population abundance	For MACS sorting, post-sort cells were at least 95% pure for CD8 T cells.
Gating strategy	FSC/SSC were gated based on cell sizes. Posive/negative boundaries were determined by comparing with an FMO (fluoresent minus one) control. A sample gating strategy is provided in supplementary figure.

- [x] Tick this box to confirm that a figure exemplifying the gating strategy is provided in the Supplementary Information.
