# Supplementary material for: DENR controls JAK2 translation to induce PD-L1 expression for tumor immune evasion
Source: Nat Commun. 2022 Apr 19;13:2059. doi: 10.1038/s41467-022-29754-y (PMC9018773; doi:10.1038/s41467-022-29754-y)
Supplement: Supplementary file 4 — Description of Additional Supplementary Files [file 41467_2022_29754_MOESM4_ESM.pdf]

Title: Supplementary Data 1.
Description: sgRNA sequences used in CRISPR screen.
